# Intestinal Obstruction Unraveled: A Rare Case of Primary Sclerosing Encapsulating Peritonitis

**DOI:** 10.7759/cureus.42289

**Published:** 2023-07-22

**Authors:** Bashar Jarrad, Laith A Ayasa, Mohammed B Abboushi, Khaled A Judeh, Nadeem Almasry, Kamal A Hamayel, Abdellatif Khader

**Affiliations:** 1 General Surgery, Palestine Medical Complex, Ramallah, PSE; 2 Internal Medicine, Al-Quds University, Jerusalem, PSE; 3 Internal Medicine, An Najah National University Faculty of Medicine, Nablus, PSE

**Keywords:** idiopathic, acute abdomen, cocoon syndrome, sclerosing encapsulation peritonitis, intestinal obstruction

## Abstract

Primary sclerosing encapsulating peritonitis (PSEP), also known as abdominal cocoon syndrome, is a rare condition characterized by small bowel encapsulation by a fibrous membrane or a cocoon-like sac. It is an uncommon cause of intestinal obstruction, as less than 300 cases have been reported from all over the world. We present the case of a 57-year-old male patient who presented with acute abdominal pain, nausea, vomiting, and constipation. A trial of conservative management failed, which warranted surgical intervention. Adhesiolysis was done, resulting in the relief of the intestinal obstruction caused by cocoon syndrome. The patient experienced excellent clinical improvement postoperatively and remained symptom-free during follow-up.

Primary sclerosing encapsulating peritonitis poses a diagnostic challenge due to its rarity and nonspecific clinical presentation. A high index of suspicion, a thorough history review, a physical examination, and imaging studies are crucial for an accurate diagnosis. This case report emphasizes the importance of recognizing abdominal cocoon syndrome as a potential cause of intestinal obstruction and highlights the successful management of the condition. This is the first case of such a disease entity to be reported from Palestine.

## Introduction

Sclerosing encapsulating peritonitis (SEP) is a rare disease characterized by intestinal obstruction due to the small bowel being matted together by a fibrocollagenous membrane (cocoon-like sac) [1]. This condition can be classified into primary (or idiopathic) sclerosing encapsulating peritonitis (PSEP), also known as an abdominal cocoon, or secondary sclerosing encapsulating peritonitis [2]. Cocoon syndrome is reported to affect mostly males [3]. It can also be further categorized into three different types according to the extent of the disease [4].

Cocoon syndrome is usually diagnosed intraoperatively due to the limitations posed by laboratory and imaging modalities [5]. There are no clear guidelines regarding the management of SEP. However, surgical intervention, including excision of the sac, adhesiolysis, and prophylactic appendectomy, gives a good clinical outcome [1].

In this paper, we report the case of a 57-year-old male who presented with features of intestinal obstruction due to an abdominal cocoon and was successfully managed with surgical intervention. This is the first case of such an entity to be diagnosed in Palestine.

## Case presentation

A 57-year-old male patient with an unremarkable past medical and surgical history presented to our emergency department complaining of abdominal pain in the periumbilical area associated with nausea, vomiting, and constipation for two days. There was no history of fever, chills, blood per rectum, or urinary symptoms. Over the past year, the patient reported similar attacks, for which he never sought medical treatment due to spontaneous symptomatic relief. The patient had had no abdominal surgeries. He had a known case of a congenital solitary kidney.

The patient’s vital signs were stable; they showed a blood pressure (BP) of 125/78 mmHg, a pulse of 72 beats per minute, an oxygen saturation of 97%, and a temperature of 36.9°C. On physical examination, the abdomen was soft but severely tender in the lower quadrants. There were no signs suggesting peritonitis. A digital rectal examination revealed an empty rectum without any fissures, hemorrhoids, or signs of bleeding. Basic blood workups were unremarkable.

**Figure 1:**
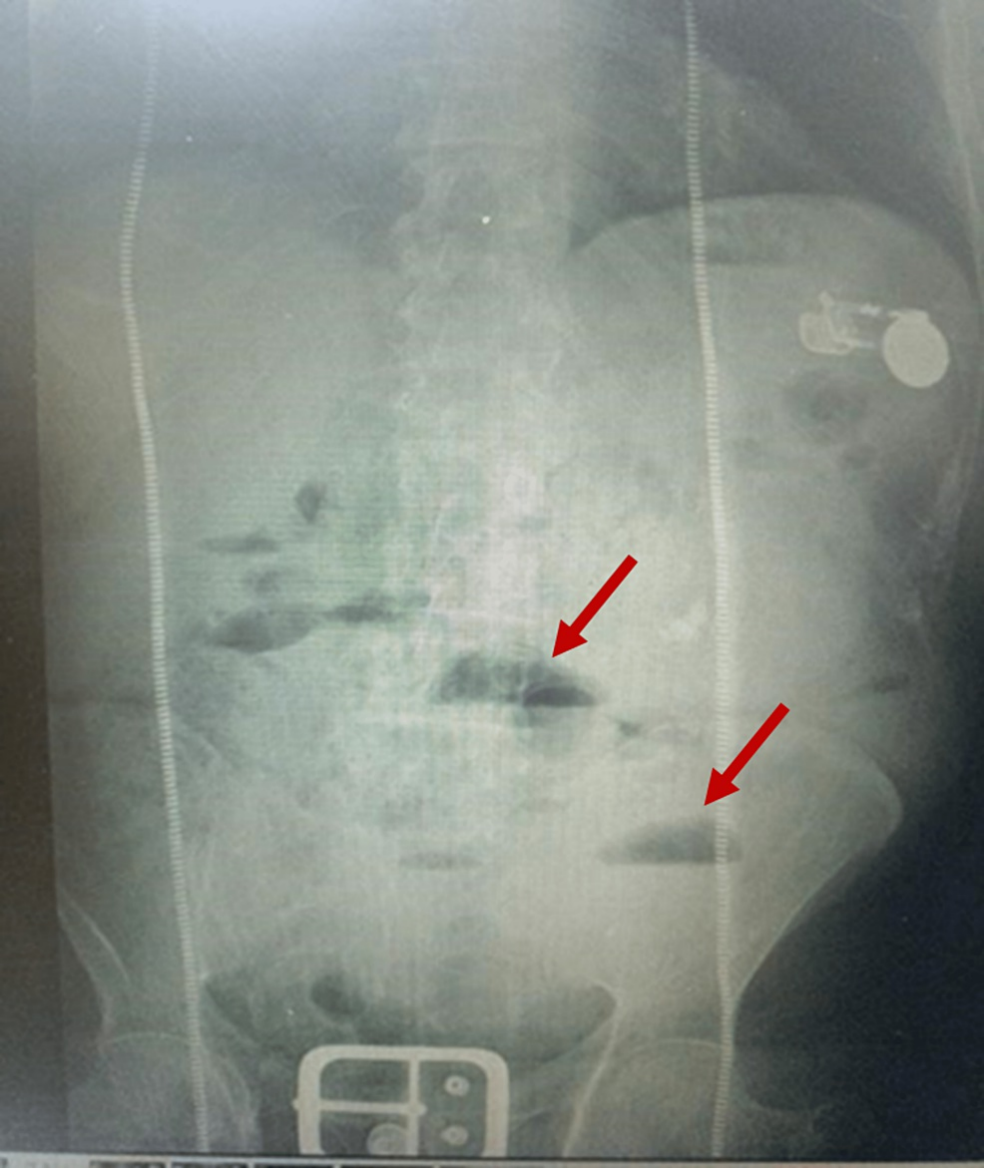
A plain abdominal X-ray revealed multiple air-fluid levels (red arrows) with dilated small bowel loops, suggestive of intestinal obstruction.

A plain abdominal X-ray was done and revealed multiple air-fluid levels with dilated small bowel loops, suggestive of intestinal obstruction (Figure 1).

**Figure 2:**
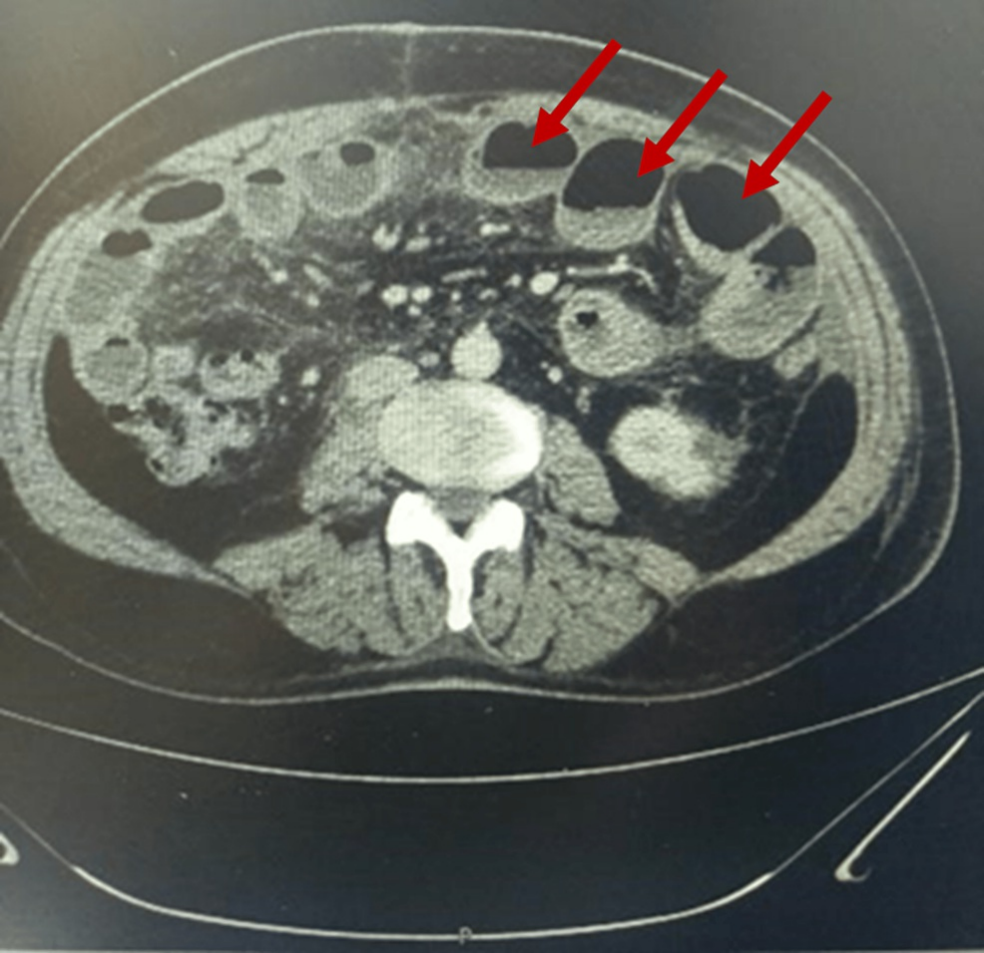
An axial cut of an abdominal CT scan showed small bowel dilatation with air-fluid levels (red arrows) and loops proximal to collapsed loops.

**Figure 3:**
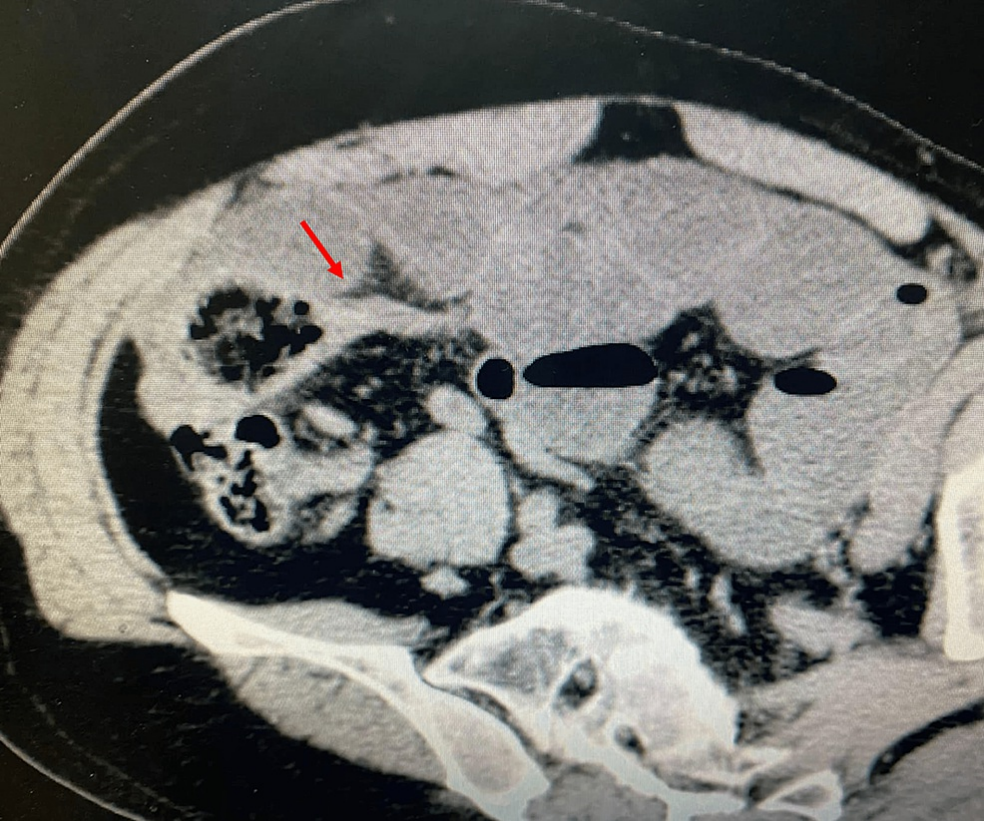
An axial cut of an abdominal CT scan also showed a transition zone in the small bowel (red arrow).

A subsequent abdominal contrast-enhanced computed tomography (CECT) was ordered, and it showed dilated small bowel loops (Figure 2) with a transition zone (Figure 3).

Imaging did not reveal signs of possible complications such as ischemia or viscus perforation. These findings were consistent with small-bowel obstruction.

**Figure 4:**
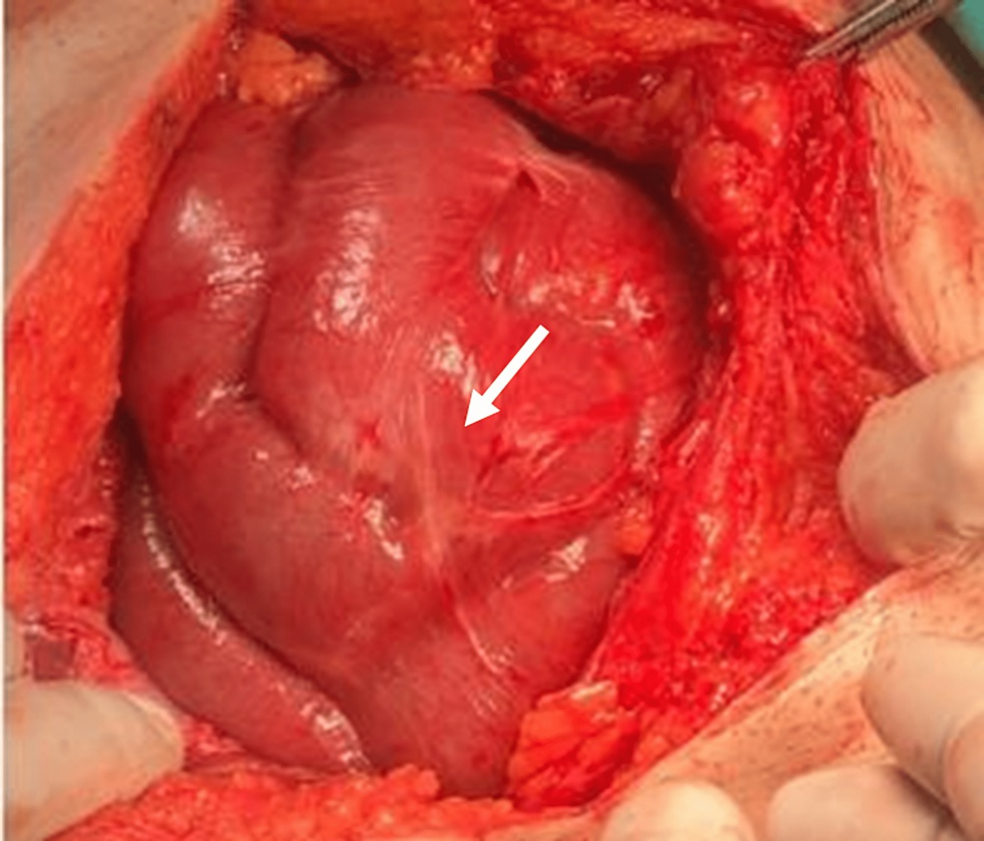
Laparotomy incision showing a thick fibrous membrane surrounding the small bowel (white arrow).

The patient was admitted for conservative management for three days, during which the patient was kept nil per os (NPO) on a nasogastric tube (NGT). He was also administered intravenous fluids. His presenting symptoms didn’t improve, and his abdomen became severely tender. The patient didn't pass stool. A blood workup revealed white blood cells (WBCs) of 17 k/uL, neutrophils (NE) of 88%, and a C-reactive protein (CRP) plasma level of 350 mg/L, so an exploratory laparotomy through a midline incision was performed, which revealed the presence of a membrane-like fibrous material encapsulating the small intestines with extensive multiple loop adhesions (Figure 4).

An extensive adhesiolysis was completed, where the membrane was excised from the small bowel to the root of the mesentery. The intestinal obstruction was relieved after surgery, and the entire small intestine was viable without serosal tears. Postoperatively, the patient displayed excellent clinical improvement. He was discharged home and was tolerating oral intake and passing stool normally. He was last seen in the clinic three months after the surgery; he was symptom-free and his wound had healed very well.

## Discussion

Sclerosing encapsulating peritonitis (SEP) is a rare disease entity characterized by the presence of a thick fibrocollagenous membrane that is partially or totally encasing the small intestines or internal abdominal structures such as the liver and stomach [1]. Sclerosing encapsulating peritonitis can be further classified based on its cause and development as either primary or secondary (acquired) [2]. The former is also termed "idiopathic SEP" or abdominal cocoon syndrome, and they usually refer to SEP in the absence of a clear cause to explain the resulting clinical manifestations following a thorough clinical, radiological, and histopathological examination [4]. The idiopathic form is even more rare than the acquired one [6]. According to Danford et al., the prevalence of SEP is yet to be determined due to its infrequency [7]. Various reports have highlighted the rarity of PSEP, documenting around 140 cases in China and India, six cases in Africa [1], and only eight cases in the Gulf region [8]. Another 2015 systematic review consisting of 193 PSEP cases revealed that it is twice as common in males than females [1], contrary to what was reported before as being most prevalent amongst young females due to the suggested pathophysiological role of retrograde menstruation, leading to the formation of a fibrous sheath [8]. Li et al. and Wei et al. also reported 66 (out of 89) PSEP cases in male patients [9,10]. These recent reports debunk the retrograde menstruation theory and reiterate that there are still no identifiable causes for PSEP, although a significant role for cytokines, fibroblasts, and angiogenic factors has been suggested [11].

The clinical presentation in such cases is variable and can present in an acute, subacute, or chronic manner [12]. As evident per our case, the majority of PSEP patients presenting acutely to the hospital would complain of signs and symptoms concerning intestinal obstruction due to compression and kinking of the bowel loops within the cocoon [12]; this includes abdominal pain and distention usually associated with nausea and vomiting [2]. These symptoms can manifest in an episodic nature with no symptoms in between, further supporting the diagnosis [13]. The presence of an abdominal mass has been mentioned in some cases as well [14]. Primary sclerosing encapsulating peritonitis cases presenting in a chronic manner can manifest as weight loss and malnutrition [9].

Due to the lack of specificity of signs and symptoms, the diagnosis of PSEP requires a high index of suspicion following a thorough revision of the medical and surgical history, a comprehensive physical examination, laboratory studies, and imaging to rule out the most common causes that might result in intestinal obstruction, as up to 80% of the cases are caused by intraabdominal adhesions. According to a large case series conducted in India and China, more than half of patients with PSEP get diagnosed intraoperatively, revealing a thickened brownish peritoneum, adherent loops of bowel, peritoneal calcifications, and a cocoon-like fibrous membrane encasing parts of the small intestines, while only 16% of patients get diagnosed preoperatively via the different imaging modalities available [7]. Despite the lack of reported cases and consensus on the best diagnostic and therapeutic approach for PSEP, contrast-enhanced computed tomography (CECT) scanning seems to be the modality of choice [2]. The key findings that appear on CT include matted, dilated bowel segments with a thick peritoneal membrane (>2 mm), adhesions, and ascitic fluid between bowel loops [15]. Other findings reported include the bottle gourd sign (a dilated second and third part of the duodenum with encasement of the distal duodenum and proximal jejunum) [16], the smudged appearance of the greater omentum [17], and focal or diffuse calcifications on the membrane or lymph nodes. Contrast CT scans have also been deemed useful in detecting complications, including bowel ischemia (or strangulation), manifested as a lack of bowel enhancement as well as perforation. Additionally, there are no specific biomarkers or laboratory findings other than nonspecific variables linked to infection and inflammation, such as inflammatory cytokines [18]. In cases of secondary SEP, the workup would consist of trying to identify the predisposing diseases such as peritoneal dialysis (PD)-related conditions, abdominal tuberculosis, sarcoidosis, endometriosis, drug-related causes such as chemotherapy, and many more underlying causal factors [2].

As performed for our patient, a laparotomy followed by membrane excision and adhesiolysis is considered the standard approach to managing patients presenting with intestinal obstruction due to PSEP [19]. A trial of conservative management consisting of bowel rest, decompression, and nutritional support can be considered in stable patients [2]. Some of the most feared postoperative complications include intestinal obstruction, infection, fistula formation, or even perforation [9].

## Conclusions

Abdominal cocoon syndrome, also known as primary sclerosing encapsulating peritonitis (PSEP), is a remarkably rare disease characterized by the presence of a fibrocollagenous membrane encapsulating the small intestines or internal abdominal structures. Due to its rarity, the diagnosis of PSEP requires a high index of suspicion and is often confirmed intraoperatively, although contrast-enhanced CT scans can aid in the diagnosis.

Our case highlights the importance of considering PSEP as part of the differential in cases of episodic bowel obstruction after ruling out the other most commonly encountered causes. The standard approach to managing PSEP with intestinal obstruction is surgical intervention, including laparotomy, membrane excision, and adhesiolysis. Conservative management may be considered for stable patients.
